# Editorial: Gut-microbiota-brain axis in depression: mechanisms and possible therapies

**DOI:** 10.3389/fnbeh.2023.1221141

**Published:** 2023-06-06

**Authors:** Ana Paula Pesarico, Angelica Thomaz Vieira, Suzan Gonçalves Rosa

**Affiliations:** ^1^Universidade Federal do Pampa - Campus Uruguaiana, Uruguaiana, Brazil; ^2^Laboratory of Microbiota and Immunomodulation, Department of Biochemistry and Immunology, Institute of Biological Sciences, Federal University of Minas Gerais, Belo Horizonte, Brazil

**Keywords:** microbiota, depression, brain, probiotics, microRNAs

Some researchers have pointed out that changes in the gut microbiota, a complex ecosystem, that begins to colonize human intestines immediately after birth, would lead to a systemic alteration. These alterations that in different ways would reach the central nervous system modulating pathways, such as the inflammatory pathway, especially the microglia, which could influence responses to treatments to depression (Macedo et al., 2017; Zhang et al., 2017; Ma et al., 2022).

Depression is a psychiatric condition that affects a large number of people in the world, but its treatment is not effective for all individuals affected (World Health Organization, 2017). Thus, this Research Topic collected current knowledge and advanced studies on the role of intestinal microbiota in depression and discussed the treatments that alter the gut microbiota.

The first review published in this Research Topic by Rosa et al. discusses the potential roles of microbiota and MicroRNAs (miRNAs) on the neuropathology of depression and anxiety, and its potential as treatment strategies. The miRNAs are small non-coding ribonucleic acid (RNAs), with an average of 22 nucleotides in length, which function as the posttranscriptional regulators of gene expression, primarily through translational repression (Ha and Kim, 2014). Studies have suggested miRNAs as pharmacological targets and biomarkers for treating and diagnosing depression and anxiety (Ortega et al., 2021; Hassan et al., 2022). Taking into account some reviews and original articles, the authors suggest that gut microbiota can influence miRNAs expression in different brain regions related to depression and anxiety, suggesting the potential role of specific miRNAs as an emerging treatment for neuropsychiatric disorders.

On the other hand, Forth et al. used current literature to demonstrate that probiotic or synbiotic supplementation alleviates symptoms of some severe psychiatric disorders, such as major depressive disorder. Probiotics and symbiotic (combine probiotics and prebiotics), when administered in adequate amounts, provide health benefits on the host and may contain a variety of microorganisms (Johnson et al., 2021). Interestingly, probiotics or fecal microbiota transplants from healthy patients alleviate symptoms and induce positive outcomes in patients with psychiatric disorders (Chinna Meyyappan et al., 2020; Johnson et al., 2021).

The authors selected 8 papers to discuss the review, because it's relevant. Some articles suggest that probiotic and symbiotic adjuvant treatment with selective serotonin reuptake inhibitors is more effective in decreasing depressive symptomatology than selective serotonin reuptake inhibitors treatment alone. In contrast, for individuals with schizophrenia, adjuvant probiotic treatment was not found to be more effective in reducing clinical symptom severity than standard antipsychotic treatment alone, but was associated with a decrease of adverse events and side effects. In the end, the authors conclude there are some big limitations, such as the small number of studies for each psychiatric illness and the lack of studies investigating other psychiatric illnesses and the mechanisms of action for these beneficial effects of probiotic adjuvant treatment are not fully understood.

Another interesting study that is part of this Research Topic is an original article by Shimada et al. exploring the relationship between social isolation-induced depressive-like phenotypes, the microbiota and liver metabolism. To this, the authors established a model of weak depression, because this type of depression is more common in modern society compared to widely studied models of severe depression (Cho et al., 2019).

Studies have been demonstrated that both microbiological and physical interaction with the mother are critical for maintaining the offspring's mental health (Benner et al., 2014; Tochitani et al., 2016). The data of Shimada et al. show that single housing conditions induce weak depression-like behaviors and demonstrate that changes in the gut bacteria composition affect the behavior and metabolism of animals.

The central mechanism for these changes induced by social isolation appears to be associated with suppression of cAMP signals in the amygdala and decrease of beta-oxidation in the liver. In this context, studies have indicated the essential role of cAMP signaling to neural response to stressors in the amygdala (Tronson et al., 2012; Cowansage et al., 2013). Interestingly, the study by Shimada et al. showed a correlation of gene expression levels with the occupation of multiple genera of intestinal bacteria, such as lactobacillus and an aero stipes, and carnitine palmitoyl transferase 1A in the liver, a key beta-oxidation enzyme. Taken together, these data suggest the essential role of gut bacteria in brain and liver homeostasis. In this way, this article provides a suitable model study of weak depression, in addition to providing therapeutic targets such as the intestinal microbiota involved with this disease and the cAMP pathway.

Studies have shown that the modulation of the intestinal microbiota can improve depression symptoms (Messaoudi et al., 2011; Codagnone et al., 2019). In this context, literature data show the effect of the intestinal microbiota on the levels of neurotransmitters involved in the mechanisms of action of antidepressants (Stasi et al., 2019). Contributing to this field of research, Rukavishnikov et al. investigated the effect of antidepressants on normal gut microbiota *in vitro* and the consequences of their antibacterial effect on treatment outcomes. The results of their study showed that selective serotonin reuptake inhibitors, selective serotonin and noradrenaline reuptake inhibitors and noradrenergic and specific serotonergic antidepressant had an inhibitory effect on the growth of all studied microorganisms.

Interestingly, the pharmacological class of antidepressant drugs studied by Rukavishnikov et al. are most often used in clinical practice for major depression treatment. In addition, the bacterial strains used in this study continued the normal human gut microbiota and played an important role in the functioning of the host organism. This study suggests that the antidepressants affect the gut microbiota and could influence the therapeutic process. Despite the limitation of the *in vitro* methodology, Rukavishnikov et al. reveals new directions for optimizing the personalized therapy of patients with depression, considering individual microbiome profiles and antidepressant used in the treatment.

In conclusion, preclinical and clinical studies that show the treatment options that modify the gut microbiota, including, for example, prebiotics and probiotics, are important to science and could contribute to finding new treatments for depression.

## Author contributions

All authors listed have made a substantial, direct, and intellectual contribution to the work and approved it for publication.
